# Rib and pericardium invaded huge abdominal mass in young woman: A case report with literature review

**DOI:** 10.1097/MD.0000000000030371

**Published:** 2022-09-02

**Authors:** Han Wool Park, Jae Hyuk Do, Tae Young Park, Hyoung-Chul Oh, Joong-Min Park, Soon Auck Hong, Hyun Jeong Park

**Affiliations:** a Division of Gastroenterology, Chung-Ang University College of Medicine, Seoul, Republic of Korea; b Department of General Surgery, Chung-Ang University College of Medicine, Seoul, Korea; c Department of Pathology, Chung-Ang University College of Medicine, Seoul, Korea; d Department of Radiology, Chung-Ang University College of Medicine, Seoul, Korea

**Keywords:** bone invasion, Desmoid fibromatosis, huge mass, pericardium invasion, surgical resection

## Abstract

Desmoid fibromatosis is a rare benign tumor, but due to its rarity and diverse clinical course, treatment guidelines have not been established. However, since a good prognosis can be expected, an accurate diagnosis and appropriate treatment are required. We describe a rare case of desmoid fibromatosis on young female that presented as huge abdominal mass.

**Patients concerns:**

A 28-year-old female with left upper abdominal pain 1 month ago was referred.

**Diagnoses:**

Abdominal computed tomography and magnetic resonance imaging revealed a heterogeneous soft tissue mass approximately 29 × 17 cm in size in the left abdomen with abdominal wall invasion and pathological fracture in costochondral junction of the left 8th to 10th ribs.

**Interventions:**

Surgical resection was performed.

**Outcomes:**

33 × 23 × 6 cm sized tumorous mass showed proliferation of bland fibromatosis and myofibroblast with nuclear β-catenin expression on pathological examination. Desmoid fibromatosis arising from intra-abdominal soft tissue with ribs and pericardium invasion was diagnosed.

**Lessons:**

The mainstay of treatment of symptomatic desmoid fibromatosis is surgical resection, and in the case of abdominal tumor, it can be more dangerous when it invades adjacent organ. We report a case that required additionally multidisciplinary approach for surgery and postoperative treatment of huge abdominal desmoid tumor which infiltrate bone and pericardium beyond abdominal cavity.

## 1. Introduction

Desmoid fibromatosis, also known as desmoid tumor, is a benign, uncommon neoplasm but frequently aggressive tumor of originated from myelofibroblast.^[1]^ It has an incidence of 0.03% of all neoplasms and <3% of all soft tissue tumors.^[2]^ Despite of its nature as benign tumor, desmoid tumor can develop as a result of progressive and aggressive behavior. Therefore, clinical management of patients with desmoid tumor is difficult for clinician. Clinical suspicion, early detection and curative resection of surgical method is important for treatment of desmoid tumor. Herein, we report a case of desmoid tumor which invaded to ribs and pericardium in young female with huge abdominal mass.

## 2. Case report

**Figure 1.**
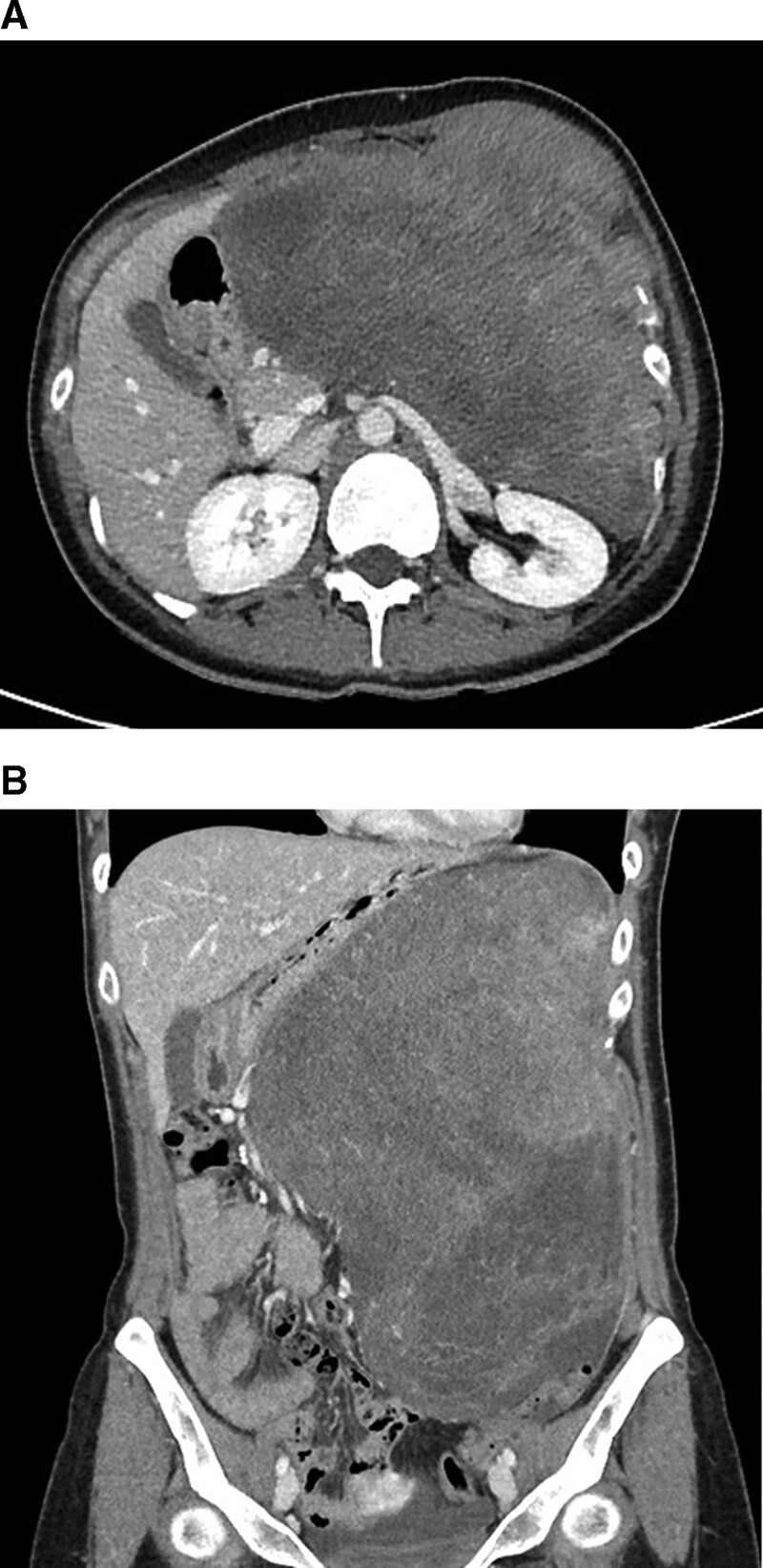
Contrast-enhanced abdominal computed tomography. (A) Transverse view of contrast-enhanced abdominal CT. (B) Coronal view of contrast-enhanced abdominal CT. About 29 × 17cm heterogeneous soft tissue mass in left abdomen with invasion to left anterior abdominal wall and costochondral junction of left 8th to 10th ribs resulting pathological fracture.

**Figure 2.**
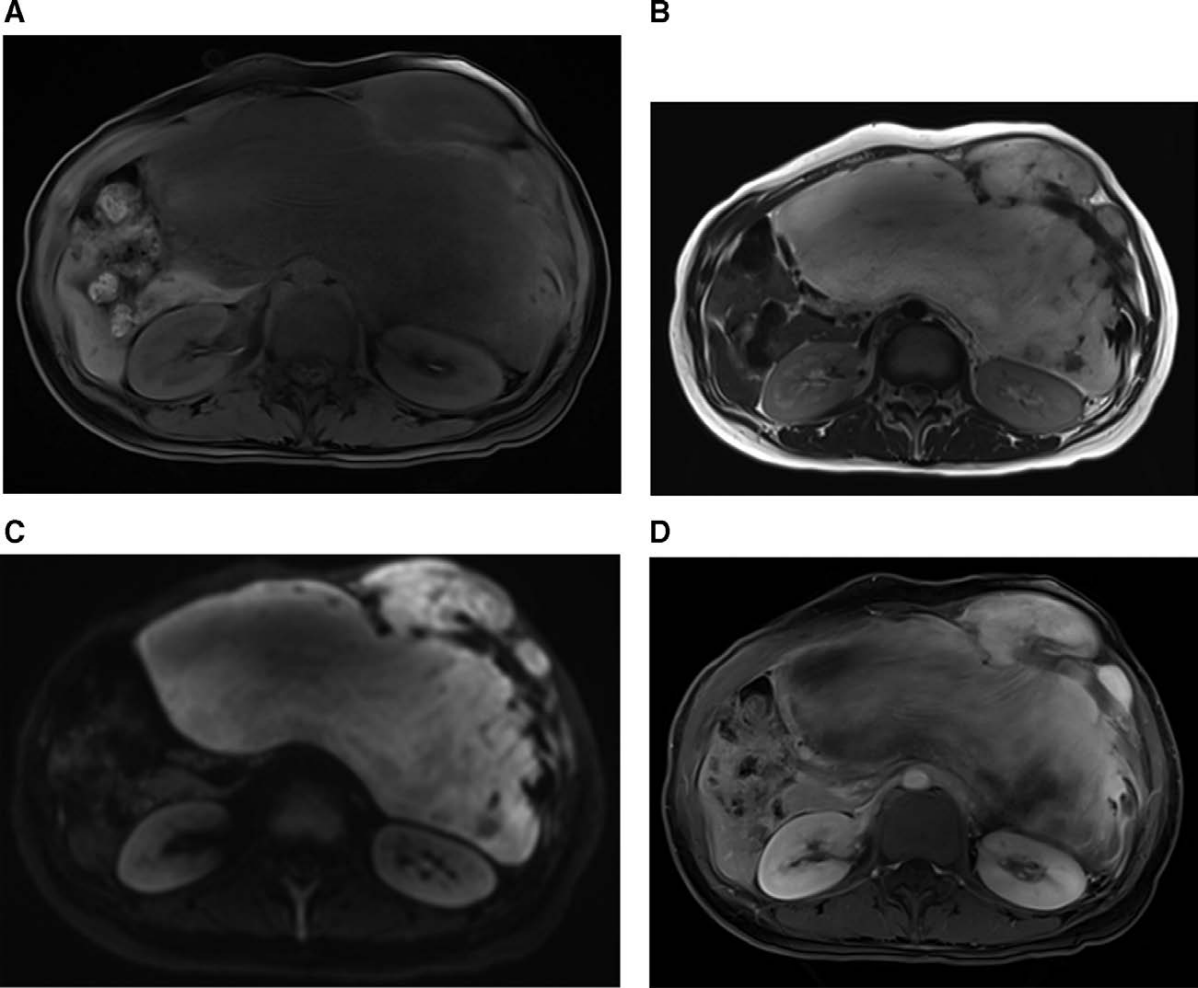
Diffusion-weighted and contrast-enhanced abdominal magnetic resonance imaging. (A) T1 weighted MRI image. The mass showed that low signal intensity with abdominal wall and left 8th to 10th ribs invasion. (B) T2 weighted MRI image. The mass showed that bright signal intensity and heterogeneous component. (C) Diffusion-weighted MRI image. The mass on abdominal wall showed focal bright signal intensity, but heterogenous and low signal intensity was noted on intra-abdominal component of mass. (D) T1 weighted MRI image on 3 minutes delayed phase. Bright but heterogenous signal intensity with gradual and slow enhancement was noted.

A 28-year-old female was referred to our hospital with left upper abdominal pain for 2 months. She went to local clinic because of her symptom, and she was performed abdominal ultrasonography (US). Under US, huge mass was found. On admission to our hospital, she had palpable mass on left side abdomen. She had no specific past medical history or obstetric history. On physical examination, a huge, fixed and hard mass with tenderness was palpable throughout the left side whole abdomen. Blood tests showed unremarkable results (white blood cell count, 7140/μL; red blood cell count, 4.63 × 10^6^/μL; hemoglobin, 120 g/dL; hematocrit, 39.4%; platelets, 278 × 10^6^/μL; C-reactive protein, 0.3 mg/L; lactate dehydrogenase, 134 IU/L). Contrast-enhanced abdominal computed tomography (CT) was showed 29 × 17 cm sized heterogeneous soft tissue mass in left abdomen. It was accompanied with left anterior abdominal wall invasion with pathological fractures in costochondral junction of left 8th to 10th ribs (Fig. 1). Diffusion-weighted and contrast-enhanced abdominal magnetic resonance imaging was showed similar finding to CT; about 29 × 17 cm malignant looking mass in left abdomen with invasion to abdominal wall and left 8th to 10th ribs with pathological fractures (Fig. 2).

Based on these findings, it was suggested that differential diagnosis for mesenchymal origin tumor such as sarcoma, gastrointestinal stromal tumor, or carcinoid tumor should be necessary. Therefore, surgery was performed due to confirmative diagnosis and treatment. In operation, huge tumorous mass invading to the left anterior abdominal wall, diaphragm, pericardium, and left 8th to 10th ribs were noted. We proceeded to a total resection of the mass invading tissue including abdominal wall, ribs, diaphragm, and pericardium, and the defect repair of pericardium, diaphragm, and peritoneal wall were performed with a composition mesh.

**Figure 3.**
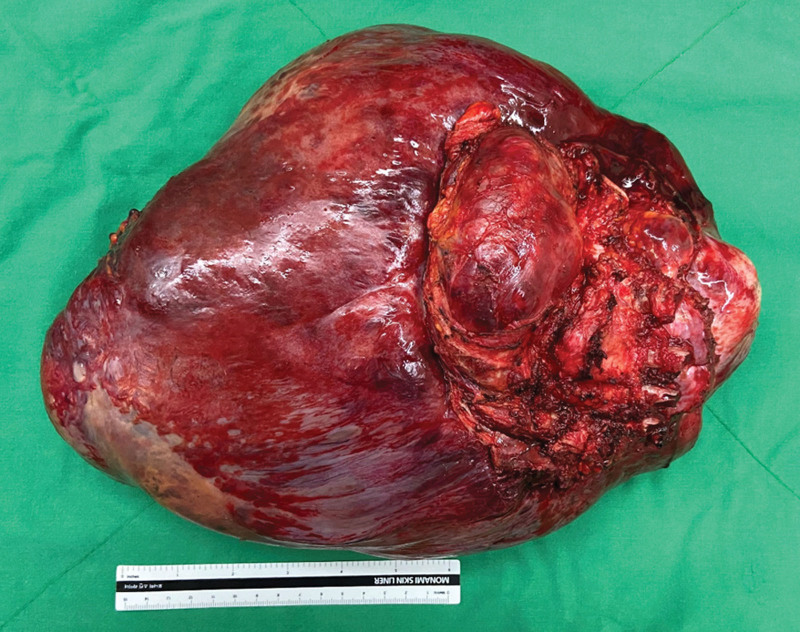
Specimen obtained during surgery. The size of the tumor was measured to be 33 × 23 × 6 cm, and macroscopically had a firm texture with hemorrhagic and necrotic appearance on the cut surface.

**Figure 4.**
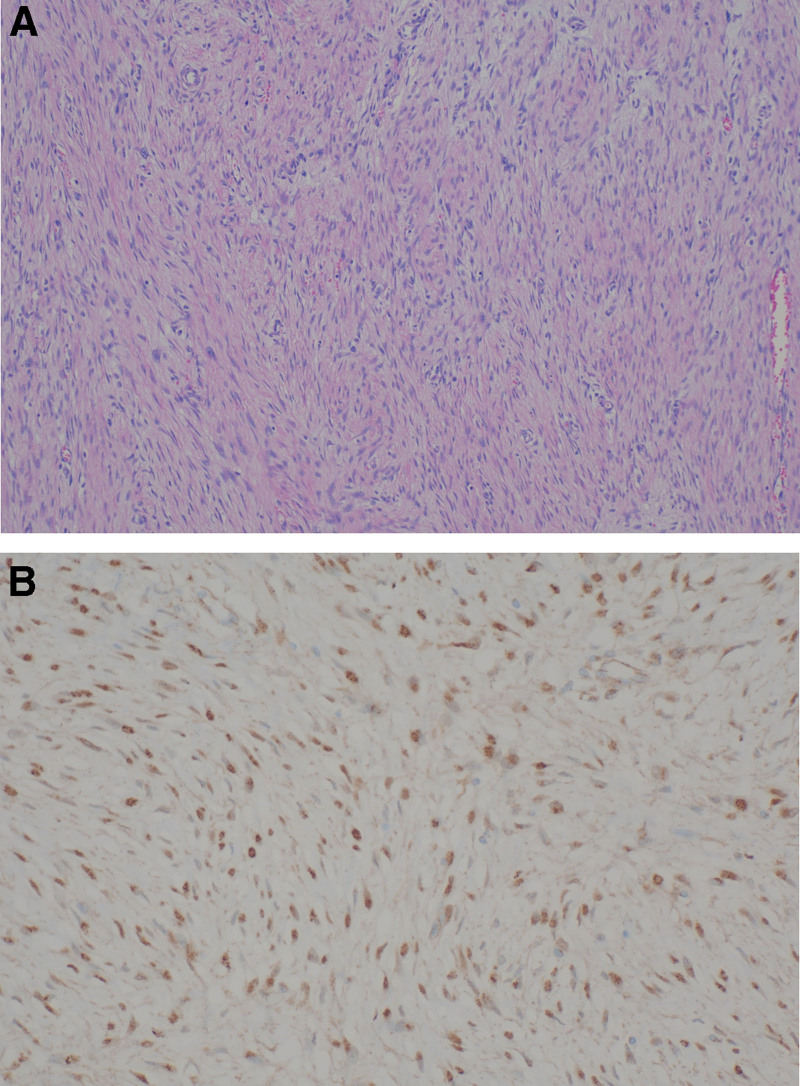
Pathologic examination of specimen. (A) Hematoxylin and eosin staining image (original magnification ×200). Microscopic examination shows the proliferation of fibroblast with a fascicular pattern. (B) Immunohistochemical staining image for β-catenin. Tumor cells showed β-catenin nuclear expression by immunohistochemistry.

The size of the tumor was measured to be 33 × 23 × 6 cm, and macroscopically had a firm texture with hemorrhagic and necrotic appearance on the cut surface (Fig 3). Pathological examination showed the proliferation of bland fibroblasts and myofibroblast without hyperchromasia of nucleus or cytologic atypia (Fig. 4A). On immunohistochemical staining, tumor cells revealed nuclear β-catenin expression (Fig. 4B). Finally, the tumor was diagnosed as desmoid fibromatosis through these pathological findings. The postoperative course was uneventful. Patient was discharged well 6 days after surgery.

Because of positive resection margin after operation, evaluation of necessity for additional adjuvant therapy for remained tumor was performed. postoperative chest, abdominal CT and positron emission tomography-computed tomography (PET-CT) was performed. On chest, abdominal CT and PET-CT, there was no pathological findings for remained tumor. Adjuvant radiation therapy was planned. The patient has provided informed consent for publication of the case. The study was approved by the Institutional Review Board of the Chung-Ang University Hospital (No. 2022-0088).

## 3. Discussion

Desmoid fibromatosis is uncommon neoplasm comprised of fibroblastic proliferations that arise in the deep soft tissues.^[3]^ It is classified as benign neoplasms due to their histopathological features and nonmetastatic properties, but show invasive growth and local infiltration. Desmoid fibromatosis occur between the age of 15–60 years, and more commonly observed in women than in men (2:1–5:1).^[4]^ Depending on the tumor site, divided into abdominal or extra-abdominal disease, and the abdominal desmoid fibromatosis is the most common type, which is associated with familial adenomatous polyposis or abdominal surgery.^[1]^

Although desmoid fibromatosis has low morbidity rate, can have potential for mortality due to their ability to invade adjacent critical organs and structures. Thus, desmoid fibromatosis shows various clinical course according to the tumor growth pattern, size, location, and organ invasion. In case of abdominal desmoids, surgery can be much more dangerous than nonabdominal desmoids, due to hemorrhagic complications or extensive enterectomy.

Because of these features of “benign malignancy,” management is difficult and many issues remain controversial. Traditionally, marginal negative resection was the standard of the treatment, but it has not proven an effect of microscopic margins on recurrence. Efforts for marginal negative can lead to unnecessary morbidity and do not reliably prevent local recurrence. Thus, the mainstay of surgical therapy should be focused on preserving function and structure while keeping the resection margin as clear as possible.

In our case, patient had a huge tumor size accompanied by organ invasion including ribs and pericardium. Among intra-abdominal desmoid fibromatosis, vigorous invasion to the adjacent organs is rare, but there are cases reported (Table 1). We found 10 huge abdominal desmoid tumor cases, and 6 of them showed adjacent organ invasion, most of which were confined to the abdominal cavity. Bone invasion occurred in only 1 case, and our case showed not only bone but also pericardial involvement, which is first reported here. We planned the radiation therapy after surgery according the results of many studies, which showed that recurrence rate dropped from 59 to 25% when radiation therapy added to operation in case of positive margin.^[5]^

**Table 1:** Characteristics of cases of large abdominal/abdominal wall desmoid tumor.

Author, year	Sex/Age	Symptom	Tumor size	Origin	Invasion	Treatment	Margin involvement	Follow-up/outcome
Mabrouk 2011^[6]^	F/41	Painful mass	10.9 × 5.9 × 7.6 cm	Abdominal wall	Rectus abdominis muscle, iliac crest, iliac spine, greater omentum, sigmoid colon	Surgery	(−)	1 yr/CR
Economou, 2011^[7]^	M/40	Painless mass	9 × 8 × 6 cm	Rectus abdominis muscle	None	Surgery	(−)	3 yrs/CR
Trigui K, 2013^[8]^	F/28	Painless mass	23 × 18 × 13 cm	Abdominal wall	None	Surgery	(−)	3 yrs/CR
Ma, 2013^[9]^	F/17	Painless mass	9.3 × 6.1 cm	Abdominal wall	None	Surgery	(−)	5 mo/CR
Palladino, 2014^[10]^	M/69	PTE	20 × 11 × 16 cm	Mesentery	Jejunum, ileocolic anastomosis	Surgery	(−)	Died on POD no. 1
Kovačević, 2017^[11]^	F/28	Abdominal pain	18 × 14 × 11 cm	Not specific-intra-abdominal soft tissue	Spleen, left adrenal gland, stomach, pancreas, hemidiaphragm, transverse colon	Surgery	(−)	Not available
Ekinci, 2017^[12]^	F/19	Painless mass	37 × 26 × 12 cm	Pancreas	Spleen	Surgery	(−)	Not available
Mahnashi, 2020^[13]^	M/48	Abdominal pain	15.8 × 14.6 × 12.8 cm	Jejunal wall	None	Surgery	(−)	Not available
Alghamdi, 2021^[14]^	M/42	Abdominal pain	26 × 17 × 9 cm	Pancreas	Transverse colon, spleen, left adrenal	Surgery	(−)	5 yrs/CR
Current case	F/28	Abdominal pain	33 × 23 × 6 cm	Mesentery	Diaphragm, pericardium, and left 8th to 10th ribs.	Surgery	(+)	Adjuvant RT, but follow-up loss

F = female, M = male, CR = complete remission, RT = radiation therapy, PTE = pulmonary thromboembolism.

Depending on the stage of the disease, various therapeutic options such as NSAIDs, antihormonal therapies, cytotoxic agents, targeted agents have been investigated.^[15]^ Church et al have proposed a staging system for intra-abdominal or trans-abdominal desmoid fibromatosis, and Latchford et al suggested therapeutic algorithm, but there are no consensus guidelines yet.^[16,17]^ Appropriate treatment can be varied from just “wait and see” to radical resection or systemic therapy, so patients with desmoid fibromatosis should be managed with multidisciplinary approach.

One of the difficult aspects of dealing with desmoid fibromatosis would be high recurrence rate. Recurrence rates following surgery of intra-abdominal desmoid fibromatosis have been reported ranged between 57% and 86% are higher than those reported for extra-abdominal tumors.^[18]^ Although most relapses occur within 3 years, it is possible even years after treatment.^[19]^ Surgery is indicated for the management of recurrences, however further local recurrence is even higher in these patients because surgical trauma itself is the risk factor for recurrence.^[19]^ Therefore, close surveillance is necessary for a sufficient period.
